# Low Dietary Bromide Intake and Sex-Specific Mortality in Kidney Transplant Recipients

**DOI:** 10.1016/j.cdnut.2026.109543

**Published:** 2026-09-25

**Authors:** Yvonne van der Veen, Caecilia SE Doorenbos, Daan Kremer, Daan J Touw, Eva Corpeleijn, Gerald Rimbach, Coby Annema, Casper FM Franssen, Stephan JL Bakker, Adrian Post

**Affiliations:** 1 Department of Internal Medicine, Division of Nephrology, University Medical Center Groningen, University of Groningen, Groningen, The Netherlands; 2 Department of Health Sciences, Division of Nursing Sciences, University Medical Center Groningen, University of Groningen, Groningen, The Netherlands; 3 Department of Dietetics, University Medical Center Groningen, University of Groningen, Groningen, The Netherlands; 4 Department of Clinical Pharmacy and Pharmacology, University Medical Center Groningen, University of Groningen, Groningen, The Netherlands; 5 Department of Epidemiology, University Medical Center Groningen, University of Groningen, Groningen, The Netherlands; 6 Division of Food Science, Institute of Human Nutrition and Food Science, University of Kiel, Kiel, Germany

**Keywords:** kidney transplant recipients, urinary bromide excretion, all-cause mortality, dietary intake, sex differences

## Abstract

**Background:**

Low bromide intake may be a modifiable risk factor for reduced survival among kidney transplant recipients (KTRs).

**Objectives:**

We investigated dietary determinants of 24-h urinary bromide excretion and examined the association between 24-h urinary bromide excretion and mortality. Given potential sex differences, additional sex-stratified analyses were performed.

**Methods:**

Adult KTRs from the TransplantLines Food & Nutrition Biobank and Cohort Study were included. Dietary determinants of urinary bromide excretion were assessed with multivariable linear regression. Associations of 24-h urinary bromide excretion were evaluated using multivariable sex-stratified Cox regression analyses.

**Results:**

The study included 693 KTRs (43% female; mean age 53 ± 13 y). Urinary bromide excretion was lower in females than in males [median: 2958 (IQR: 2343–3713)] compared with median: 3640 [IQR: 2853–4585] μg/24 h; *P* < 0.001}. Energy intake (*β* = 0.12, *P* = 0.002), animal protein intake (*β* = 0.16, *P* = 0.001), and water intake (*β* = 0.13, *P* = 0.005) were positively associated with urinary bromide excretion. In sex-stratified analyses, animal protein intake was a significant determinant of bromide excretion in females (*β* = 0.22, *P* = 0.002), whereas water (*β* = 0.15, *P* = 0.02) and energy intake (*β* = 0.13, *P* = 0.01) were associated with bromide excretion in males. During a median follow-up of 5.3 (IQR: 4.8–6.1) years, 150 KTRs (21.6%) died. Higher urinary bromide excretion was consistently associated with a lower risk of mortality, even after adjustment for potential confounders [hazard ratio [HR]: 0.67 [95% confidence interval (CI): 0.50, 0.90]; *P* = 0.008]. The association of urinary bromide excretion with mortality was significantly modified by sex (*P*_interaction_ = 0.02). Among females, there was a significant independent association between higher urinary bromide excretion and lower mortality [HR: 0.45 (95% CI: 0.29;0.72), *P* = 0.001], whereas no such association was observed in males.

**Conclusions:**

Energy, animal protein, and water intake were positively associated with urinary bromide excretion, with sex-specific differences. Lower urinary bromide excretion was independently associated with increased all-cause mortality in female, but not in male KTRs. These findings support further investigation into whether improving bromide status could reduce mortality, particularly among female KTRs.

This trial was registered at clinicaltrials.gov as NCT02811835.

## Introduction

Although kidney transplantation improves patient outcomes compared with chronic dialysis, kidney transplant recipients (KTRs) continue to experience reduced life expectancy compared with the general population [1,2]. Although several risk factors have been identified, it remains crucial to explore additional, preferably modifiable risk factors that could improve long-term outcomes in this population.

Bromide has historically been studied primarily from a toxicological perspective, with adverse effects predominantly described after excessive exposure [3]. However, more recent experimental studies have identified a physiological requirement for bromide. Bromide is a required cofactor for peroxidasin-mediated formation of sulfilimine cross-links in collagen IV, which structurally reinforce basement membranes [4,5]. In addition, eosinophil peroxidase can use bromide to generate hypobromous acid, which plays a role in host defense against multicellular parasites and certain bacterial pathogens [6,7]. In an experimental Drosophila study, dietary bromide depletion markedly reduced collagen IV sulfilimine cross-linking and resulted in developmental and basement membrane abnormalities, whereas bromide supplementation restored cross-link formation and rescued the observed phenotypes [4]. Together, these data suggest that a minimum level of bromide availability may be required to maintain physiological functions, including basement membrane integrity and host defense. This provided the biological rationale for our a priori hypothesis that relatively low bromide exposure could be associated with adverse long-term outcomes in KTRs.

Bromide is a naturally occurring halogen widely distributed in soil, water, and marine environments, and is therefore present in many plant- and animal-derived foods. Dietary exposure reflects both natural background levels and agricultural practices, as well as environmental and technological sources [3]. Population monitoring in Europe indicates that current dietary bromide intake is relatively low compared with historical levels, reflecting changes in food production and fumigation practices, where gaseous pesticides were used to treat food commodities, particularly with methyl bromide, to control insects, nematodes, rodents, fungi, and other pests [3]. After the establishment of an acceptable daily intake in 1966, subsequent evaluations of bromide eventually led to the phase out of the use of methyl bromide starting in 2005, with critical uses ceased by around 2009, with very restricted residual uses thereafter [3,8,9]. This decline raises the question of whether present-day bromide intake remains sufficient to meet potential physiological requirements, particularly if bromide contributes to structural or immunological processes. Unlike essential minerals, no recommended dietary allowance (RDA) has been established for bromide [10]. It is generally regarded as an ultratrace element, with growing but as yet inconclusive evidence for essentiality [11,12]. Regulatory authorities, such as the European Food Safety Authority (EFSA), have focused primarily on preventing excess exposure [3]. In 2025, EFSA established a tolerable daily intake and acute reference dose for bromide of 0.4 mg/kg body weight, based on endocrine and neurodevelopmental effects [3]. However, these values highlight only potential risks of excess intake, whereas data on the potential harmful effects of low bromide intake remain scarce.

Patients with advanced kidney disease are commonly exposed to dietary restrictions for a prolonged period before kidney transplantation, including restrictions in sodium, potassium, and, depending on disease stage and treatment modality, protein intake [13]. Although dietary recommendations change after transplantation, some restrictions remain recommended, and dietary habits established before transplantation may persist [13,14]. Such long-term dietary restrictions may increase the likelihood of relatively low intake of specific dietary components, making the potential health consequences of low bromide exposure particularly relevant to investigate in KTRs.

In this study, we aimed to investigate dietary intake determinants of 24-h urinary bromide excretion and investigate whether a lower 24-h urinary bromide excretion is associated with mortality in KTRs. Building on sex-specific differences in dietary patterns, and to comply with editorial recommendations for sex-specific reporting, we stratified analyses by sex to explore potential differential effects [15,16].

## Methods

### Study population and design

Data from the prospective TransplantLines Food & Nutrition Biobank and Cohort Study (clinicaltrials.gov: NCT02811835) were used, which has previously been described in detail [17]. In brief, this prospective cohort study included 707 adult KTRs with a functioning allograft, ∼1 y or longer after transplantation, who visited the outpatient clinic of the University Medical Center Groningen, Groningen, The Netherlands, between November 2008 and June 2011. All patients provided written informed consent. KTRs without data on urinary bromide excretion (*n* = 14) were excluded, resulting in 693 KTRs included in analyses. A flowchart of the study is shown in Supplemental Figure 1. The study was approved by the University Medical Center Groningen institutional review board (METc 2008/186) and adheres to the Declarations of Helsinki and Istanbul. The study results are reported in accordance with the STROBE guideline for observational studies (Supplemental Table 1) [18].

### Data collection

Baseline clinical data, including height, weight, and waist circumference, were measured during a visit to the outpatient clinic. Clinical, demographic, and transplantation-related characteristics were extracted from electronic patient records. Medication use and medical history were extracted from electronic patient records and then verified with all patients. Blood samples were taken after an 8 to 12 h fasting period before the study visit.

### Measurement of bromide status

Bromide is almost completely absorbed in the gastrointestinal tract and is primarily eliminated through the kidneys, with an elimination half-life of ∼12 d [3,19]. Given its renal elimination and long biological half-life, 24-h urinary bromide excretion provides a stable measure of longer-term dietary bromide intake [20]. All participants were instructed to collect a 24-h urine sample according to a strict protocol on the day before their visit to the outpatient clinic. Urine was collected under oil, and chlorhexidine was added as an antiseptic agent. Samples were stored at −80°C until analysis. Urinary bromide concentration was measured by inductively coupled plasma mass spectrometry (ICAP Q instrument, Thermo Fisher Scientific) at SGS Analytics. Measurements were conducted following Organization for Standardization (ISO) 17294-2: 2017-01 [21,22]. Before measurement, samples were decomposed with a mixture of nitric acid and hydrogen peroxide (4:1) using microwave pressure digestion. For calibration, a multielement standard was employed. To reduce spectroscopic interferences, detection was performed using a collision/reaction cell. Rhodium (2 μg/L) was added as the internal standard. The method showed a limit of detection of 2 μg/L and a limit of quantification of 5 μg/L. Recovery was 99.0%. Intraday and interday precision, expressed as coefficients of variation, were 1.5% and 2.6%, respectively.

### Dietary intake and physical activity

Information on dietary intake was collected using a validated semiquantitative food-frequency questionnaire (FFQ), which was linked to the Dutch food composition table (Nederlands Voedingsstoffenbestand; NEVO) 2006 to enable calculation of daily intakes of energy as well as various macronutrients and micronutrients [23]. Because NEVO does not provide bromide concentrations for food items, dietary bromide intake could not be estimated from the FFQ. The FFQ covers intakes of 177 food items during the past month, with seasonal variations taken into account. For each item, the consumption frequency was recorded in times per day, week, or month. The number of servings and portion sizes were expressed in natural units (e.g., slice of bread or apple) or common household measures (e.g., cup or spoon). The questionnaire was self-administered and filled out at home. Upon return, each questionnaire was checked for completeness by a trained researcher, and any inconsistencies were verified with the participant. Validation of the FFQ in KTRs has been described previously [17]. Dietary data were converted into daily nutrient intakes with the use of the Dutch Food Composition Table of 2006 [24].

Physical activity was assessed using the Short Questionnaire to Assess Health-Enhancing Physical Activity (SQUASH) [25]. The questionnaire was filled in by the participant before the study visit. The SQUASH is a self-administered instrument designed to capture habitual physical activity over the past week across 4 domains: commuting activities, household tasks, occupational activity, and leisure-time physical activity, including sports. Participants report the frequency (d/wk), average duration (min/d), and perceived intensity (light, moderate, vigorous) of activities within each domain. Total physical activity is calculated by multiplying frequency and duration across activities and summing the resulting minutes per week.

#### Laboratory measurements and calculations

Clinical chemistry assays, including parameters of kidney function, glucose homeostasis, inflammation, thyroid hormones, and urinary excretions of sodium, creatinine, cystatin C, and urea, were performed using routine spectrophotometric methods (Roche Diagnostics), as previously described [[26], [27], [28]]. Estimated glomerular filtration rate (eGFR) was calculated using the 2021 creatinine and cystatin C-based Chronic Kidney Disease Epidemiology Collaboration equation [29]. Diabetes mellitus was defined as hemoglobin A1c (HbA1C) ≥48 mmol/mol (6.5%), nonfasting glucose ≥1.0 mmol/l, fasting glucose ≥7.0 mmol/L, or the use of diabetes medication according to the criteria of the American Diabetes Association [30]. Hypertension was defined as systolic blood pressure ≥140 mm Hg and/or diastolic blood pressure ≥90 mm Hg, consistent with established international guidelines [31].

### Statistical analyses

All statistical analyses were performed using R Studio (version 4.4.0). For all analyses, a 2-sided *P* value < 0.05 was considered statistically significant. Baseline data were presented for the full KTR group as well as stratified for sex.

Normally distributed variables were presented as mean ± SD, skewed variables as median (IQR), and categorical data as number (valid percentage). Normality of distributions was assessed by visual inspection of histograms and Q-Q plots. Differences between males and females in baseline characteristics in KTRs were tested using an independent-sample t-test, Wilcoxon Mann–Whitney *U* test, depending on normality of the data, or Pearson’s chi-squared test.

Associations between dietary factors and urinary bromide excretion were evaluated using multivariable linear regression analyses adjusted for age, sex, and body surface area (BSA), with additional adjustment for total energy intake except when energy intake itself was the determinant. Additional analyses were performed stratified by sex to explore potential sex-specific associations. Associations of urinary bromide excretion with mortality were assessed using Cox proportional hazard models in the full KTR group as well as in female and male KTRs separately. Hazard ratios (HRs) for urinary bromide excretion were calculated per doubling. We visually assessed the proportional hazards assumption and observed modest deviations; however, time-dependent analyses did not indicate a substantial impact on the results. Therefore, we retained the standard Cox proportional hazards models for their more straightforward interpretability. Cox regression analyses were adjusted for a priori selected potential confounders based on prior literature and biological plausibility, including age, sex, BSA, eGFR, urinary protein excretion, time since transplantation, smoking, alcohol use, hypertension, and diabetes. Age, sex, smoking, alcohol use, hypertension, diabetes, and time since transplantation were included as established predictors of long-term outcomes in KTRs that may also be associated with dietary and lifestyle patterns [32,33]. BSA was included to account for differences in body size that may influence dietary intake and consequently 24-h urinary bromide excretion, whereas eGFR and urinary protein excretion were included because renal function may directly influence urinary bromide excretion. Potential effect modification by covariates was tested by including interaction terms (product terms between the covariate and exposure) to the model adjusted for age, sex, and BSA. An interaction term with a *P*_interaction_ < 0.10 was considered to indicate effect modification. To account for potential bias that could result from the exclusion of participants with missing covariates, multiple imputation using Fully Conditional Specification was performed using the “mice” package to obtain 5 imputed data sets. In line with common recommendations for robust multiple imputation, variables with a maximum of 15% missing values were considered suitable for imputation [34]. The highest proportion of missing data was observed for thyroid-stimulating hormone (TSH; 11%). The proportion of missing values for each variable is provided in Supplemental Table 2. The algorithm was run for 30 iterations, and convergence of the Markov chains was evaluated with trace plots of the mean and variance. To confirm that imputed values were biologically plausible, the distributions of the imputed values were visually investigated and compared with the distribution of the observed values. Analyses were performed in each of the data sets, and results, including 95% confidence intervals (CIs), were pooled using Rubin’s rules. Apart from the baseline table and unless otherwise stated, analyses were performed using imputed data sets. To assess the robustness of our findings, we performed several sensitivity analyses. These included additional analyses after exclusion of outliers (the highest fifth percentile) as well as analyses with additional adjustments for either energy intake, water intake, animal protein intake, thyroid function (free thyroxine and TSH), physical activity (measured with SQUASH), and 24-h urinary chloride excretion. These variables were selected based on their strong associations with urinary bromide excretion observed in Supplemental Table 3, where higher bromide excretion was consistently associated with higher energy intake, water intake, higher urinary volume, and chloride excretion, and modestly higher physical activity. Given that these factors may reflect underlying dietary patterns, hydration status, and lifestyle behaviors that could confound the association between bromide excretion and outcomes, they were considered potential confounders along the causal pathway.

Additional sensitivity analyses were performed to further assess the robustness of the association between urinary bromide excretion and mortality. These analyses included additional adjustment for renin–angiotensin–aldosterone system (RAAS) inhibitor use and protein intake. In addition, creatinine clearance was used as an alternative measure of kidney function, replacing eGFR in the primary multivariable model. The results of these additional analyses are presented in Supplemental Table 4.

## Results

### Patient characteristics

A flowchart of the study is shown in Supplemental Figure 1, and the STROBE guideline checklist for observational studies is shown in Supplemental Table 1. The patient characteristics are presented in Table 1. The study included 693 KTRs, of whom 299 (43%) were female, with a mean age of 53 ± 13 y and an eGFR of 52 ± 20 mL/min/1.73 m^2^. Urinary bromide excretion was lower in females than in males [median: 2958 (IQR: 2343–3713) compared with median: 3640 (IQR: 2853–4585) μg/24 h; *P* < 0.001]. As expected, females had a lower height, weight, BMI, and BSA as compared with males (all *P* < 0.001), whereas systolic blood pressure was slightly lower in females compared with males (134 ± 19 compared with 138 ± 16 mmHg; *P* = 0.006). Kidney function was slightly lower in females compared with males (50 ± 21 compared with 54 ± 20 mL/min/1.73 m^2^; *P* = 0.044). There were no significant sex differences in transplant characteristics, prevalence of diabetes, or use of immunosuppressive medication. Urinary protein excretion levels were lower in females [median: 0.1 (IQR: 0.0–0.3) compared with median 0.2 (IQR: 0.0–0.5) g/24 h; *P* < 0.001]. Dietary intake patterns differed substantially between sexes. Females reported lower energy and water intake compared with males (1939 ± 546 compared with 2362 ± 648 kcal; 1965 ± 520 compared with 2142 ± 599 mL; *P* < 0.001 for both). Males reported greater consumption of bread, potatoes, pasta, meat, and coffee (all *P* < 0.001). In contrast, females consumed more fruits and tea [median: 135 (IQR: 77–237) compared with median: 115 (IQR: 53–232) g/d; *P* = 0.004, and median: 250 (IQR: 125–500) compared with median: 143 (IQR: 22–371) g/d; *P* < 0.001, respectively].

**TABLE 1:** Patient characteristics of KTRs according to sex

	Total (*n* = 693)	Female (*n* = 299, 43.1%)	Male (*n* = 394, 56.9%)	*P*_difference_
Urinary bromide excretion (μg/24 h)	3308 (2597–4289)	2958 (2343–3713)	3640 (2852–4585)	<0.001
*Clinical characteristics*				
Age at visit (y)	53 ± 13	53 ± 13	53 ± 13	0.95
Height (cm)	173.5 ± 9.7	166.1 ± 7.7	179.2 ± 6.9	<0.001
Weight (kg)	80.4 ± 16.4	74.1 ± 15.9	85.1 ± 15.3	<0.001
Body surface area (m^2^)	1.9 ± 0.2	1.8 ± 0.2	2.0 ± 0.2	<0.001
Hypertension, *n* (%)	283 (41)	99 (33)	184 (47)	<0.001
Diabetes, *n* (%)	162 (23.4)	74 (24.7)	88 (22.3)	0.48
Current smoking, *n* (%)	81 (12.6)	25 (8.9)	56 (15.3)	0.015
Time after transplantation (y)	5.3 (2.0–12.0)	5.3 (2.1–12.3)	5.5 (1.9–11.4)	0.74
*Laboratory measurements*				
Plasma creatinine (μmol/L)	125 (100–161)	110 (89–145)	134 (109–170)	<0.001
Plasma cystatin C (mg/L)	1.89 ± 0.77	1.88 ± 0.80	1.91 ± 0.75	0.47
eGFR (mL/min/1.73 m^2^)	52 ± 20	50 ± 21	54 ± 20	0.044
Plasma FT3 (pmol/L)	4.9 ± 0.8	4.9 ± 0.8	4.9 ± 0.8	0.80
Plasma FT4 (pmol/L)	16.1 ± 2.9	16.3 ± 3.0	15.9 ± 2.8	0.063
Plasma TSH (mIU/L)	1.6 (1.0–2.5)	1.7 (1.1–2.6)	1.6 (1.0–2.4)	0.07
Urinary volume (mL/24 h)	2439 ± 799	2444 ± 825	2435 ± 780	0.9
Urinary protein excretion (g/24 h)	0.2 (0.0–0.4)	0.1 (0.0–0.3)	0.2 (0.0–0.5)	<0.001
Urinary chloride excretion (mmol/24 h)	147 ± 59	126 ± 51	162 ± 30	<0.001
Creatinine clearance (mL/min)	64 (46–83)	60 (41–75)	68 (48–88)	<0.001
*Medication*				
Calcineurin inhibitor, *n* (%)	397 (57.3)	168 (56.2)	229 (58.1)	0.67
Proliferation inhibitor, *n* (%)	573 (82.7)	248 (82.9)	325 (82.5)	0.96
RAAS blockade, *n* (%)	335 (48.3)	142 (40.1)	215 (54.6)	<0.001
Statin, *n* (%)	366 (52.8)	206 (52.3)	160 (53.5)	0.81
*Dietary intake*^1^				
Energy intake (kcal/d)	2180 ± 641	1939 ± 546	2362 ± 648	<0.001
Water intake (mL/d)	2066 ± 573	1965 ± 520	2142 ± 599	<0.001
Coffee (g/d)	500 (250–625)	375 (250–526)	500 (286–625)	<0.001
Tea (g/d)	250 (54–375)	250 (125–500)	143 (22–371)	<0.001
Bread (g/d)	131 (92–167)	108 (82–141)	143 (106–183)	<0.001
Vegetables (g/d)	91 (53–123)	91 (61–119)	85 (46–133)	0.23
Fruit (g/d)	123 (65–232)	135 (77–237)	115 (53–232)	0.004
Potatoes (g/d)	119 (79–165)	100 (63–146)	139 (90–197)	<0.001
Pasta (g/d)	19 (10–32)	16 (8–26)	24 (12–39)	<0.001
Rice (g/d)	15 (4–30)	15 (25–4)	15 (0–32)	0.94
Legumes (g/d)	4 (0–11)	1 (0–11)	4 (0–13)	0.059
Nuts (g/d)	0 (0–4)	1 (0–5)	0 (0–3)	0.033
Beer (g/d)	0 (0–54)	0 (0–0)	26 (0–171)	<0.001
Wine (g/d)	0 (0–20)	0 (0–29)	0 (0–20)	0.13
Cheese (g/d)	29 (15–46)	29 (46–15)	30 (15–46)	0.94
Egg (g/d)	9 (4–14)	7 (4–14)	9 (4–16)	0.14
Fish (g/d)	11 (4–21)	12 (4–19)	11 (4–22)	0.86
Soy products (g/d)	0 (0–0)	0 (0–0)	0 (0–0)	0.013
Meat products (g/d)	96 ± 39	88 ± 38	102 ± 39	<0.001
Dairy (g/d)	124 (62–145)	124 (62–145)	124 (60–145)	0.78
Protein intake (g/d)				
Plant protein intake (g/d)	31 ± 10	28 ± 9	33 ± 10	<0.001
Animal protein intake (g/d)	51 ± 15	49 ± 15	53 ± 15	0.002
*Physical activity*				
SQUASH score	5060 (1970–7772)	4470 (1800–6630)	5760 (2361–9150)	<0.001

Values are presented as mean ± SD, median (IQR), or number (percentage) as indicated.

Abbreviations: eGFR, estimated glomerular filtration rate; FT3, free triiodothyronine; FT4, free thyroxine; hs-CRP, high-sensitivity C-reactive protein; KTR, kidney transplant recipient; RAAS, renin–angiotensin–aldosterone system; SQUASH, Short QUestionnaire to ASsess Health-enhancing physical activity; TSH, thyroid-stimulating hormone.

1 Dietary intake was available in a subset of 631 KTRs.

Patient characteristics of KTRs by urinary bromide excretion (below compared with above the median), overall and by sex, are shown in Supplemental Table 3. KTRs with urinary bromide excretion above the median had a higher body size (height, weight, BSA, and BMI) and a higher prevalence of hypertension compared with those below the median. In addition, higher urinary bromide excretion was associated with higher urinary volume and urinary chloride excretion (both *P* < 0.001). Dietary intake differed substantially between groups, with higher intake of energy, water, protein (total, plant, and animal), and several food groups, including bread, potatoes, meat products, dairy, eggs, and fish, being associated with higher urinary bromide excretion. Conversely, tea intake was lower in the upper half. Physical activity levels were modestly higher in those with higher bromide excretion. These patterns were generally consistent in sex-stratified analyses, although associations with dietary intake were more pronounced in males than in females.

### Dietary determinants of urinary bromide excretion

A total of 631 of the 707 (89.3%) participants completed and returned the FFQ. In the total KTR population, multivariable linear regression analyses adjusted for sex showed that energy intake [*β* = 0.12 (95% CI: 0.04, 0.20), *P* = 0.002], total protein intake [*β* = 0.26 (95% CI: 0.13, 0.40), *P* < 0.001], animal protein intake [*β* = 0.16 (95% CI: 0.06, 0.25), *P* = 0.001], and water intake [*β* = 0.13 (95% CI: 0.04, 0.23), *P* = 0.005] were independently associated with urinary bromide excretion (Table 2). Sex-stratified analyses showed similar directions of association in females and males, although statistical significance was generally reached only in females. Specifically, the association between total protein intake and urinary bromide excretion was significant in females [*β* = 0.44 (95% CI: 0.24, 0.65), *P* < 0.001], but not in males [*β* = 0.11 (95% CI: −0.07, 0.29), *P* = 0.25], indicating a stronger relationship in females. Associations with animal protein intake followed a similar pattern [females: *β* = 0.22 (95% CI: 0.08, 0.37), *P* = 0.002; males: *β* = 0.10 (95% CI: −0.03, 0.22), *P* = 0.13]. In contrast, water [*β* = 0.15 (95% CI: 0.02, 0.028), *P* = 0.02] and energy intake [*β* = 0.13 (95% CI: 0.03, 0.24), *P* = 0.01] were significantly associated with urinary bromide excretion in males, whereas in females the association was in the same direction but did not reach statistical significance. Coffee [*β* = 0.12 (95% CI: 0.02, 0.22), *P* = 0.03] was significantly associated with urinary bromide excretion in males, but not in females.

**TABLE 2:** Linear regression analyses of 24-h urinary bromide excretion with dietary intake in KTRs

	Total (*n* = 631)		Female (*n* = 276)		Males (*n* = 355)	
	Standard *β* (95% CI)	*P* value	Standard *β* (95% CI)	*P* value	Standard *β* (95% CI)	*P* value
*Intakes*						
Energy intake	0.12 (0.04, 0.20)	0.002	0.10 (–0.01, 0.22)	0.08	0.13 (0.03, 0.24)	0.01
Protein intake	0.26 (0.13, 0.40)	<0.001	0.44 (0.24, 0.65)	<0.001	0.11 (–0.07, 0.29)	0.25
Animal protein	0.16 (0.06, 0.25)	0.001	0.22 (0.08, 0.37)	0.002	0.10 (–0.03, 0.22)	0.13
Plant protein	0.02 (–0.12, 0.15)	0.81	0.10 (–0.09, 0.30)	0.30	–0.07 (–0.25, 0.11)	0.44
Fat intake	0.01 (–0.17, 0.18)	0.97	–0.07 (–0.34, 0.20)	0.62	0.06 (–0.17, 0.29)	0.59
Carb intake	–0.12 (–0.29, 0.04)	0.13	–0.24 (–0.50, 0.02)	0.07	–0.08 (–0.28, 0.13)	0.45
Fiber intake	0.03 (–0.08, 0.14)	0.57	0.09 (–0.08, 0.26)	0.28	–0.01 (–0.14, 0.14)	0.98
Water intake	0.13 (0.04, 0.23)	0.005	0.13 (–0.01, 0.27)	0.08	0.15 (0.02, 0.28)	0.02
Coffee intake	0.07 (–0.01,0.15)	0.08	0.04 (–0.09,0.16)	0.58	0.12 (0.02, 0.22)	0.03
Tea intake	–0.05 (–0.13, 0.02)	0.17	0.01 (–0.11, 0.13)	0.91	–0.10 (–0.20, 0.01)	0.05
Dairy intake	0.08 (0.01, 0.16)	0.05	0.13 (0.01, 0.25)	0.05	0.06 (–0.05, 0.17)	0.27
Fish intake	0.03 (–0.04, 0.11)	0.41	0.03 (–0.08, 0.15)	0.57	0.03 (–0.07, 0.13)	0.58
Meat intake	0.03 (–0.05, 0.11)	0.50	0.03 (–0.09, 0.16)	0.62	0.03 (–0.08, 0.14)	0.59
Vegetables	0.04 (–0.04, 0.11)	0.37	0.12 (0.01, 0.24)	0.04	–0.02 (–0.13, 0.08)	0.68
Fruit	–0.03 (–0.11, 0.04)	0.39	–0.06 (–0.18, 0.06)	0.31	–0.01 (–0.12, 0.10)	0.85

Dietary intake was available in 631 out of 693 KTRs. All analyses were adjusted for age, sex, and body surface area, and were additionally adjusted for total energy intake except for energy intake itself.

Abbreviations: CI, confidence interval; KTR, kidney transplant recipient.

### Survival analyses

During a median follow-up of 5.3 (IQR: 4.8–6.1) years, 150 KTRs (21.6%) died, of whom 61 (41%) were females, and 89 (59%) were males. Cox regression analyses of 24-h urinary bromide excretion with mortality are shown in Table 3. In KTRs, higher urinary bromide excretion was associated with a lower risk of mortality in the crude model [HR per doubling: 0.73 (95% CI: 0.55, 0.96), *P* = 0.02]. This association persisted after multivariable adjustment for potential confounders [HR per doubling: 0.67 (95% CI: 0.50, 0.90), *P* = 0.008]. A graphical representation of the association of 24-h urinary bromide excretion is shown in Figure 1. Consistent with our a priori rationale for sex-stratified analyses, formal interaction testing in the overall (nonstratified) model confirmed significant effect modification by sex (*P*_interaction_ = 0.02). In sex-stratified analyses, this inverse association was present in females but not in males. Among females, after adjusting for potential confounders, the association of higher urinary bromide excretion remained significant [HR: 0.45 (95% CI: 0.29, 0.72), *P* = 0.001]. No association was observed in males. Apart from sex, no other effect modification was present.

**TABLE 3:** Cox regression analyses of 24-h urinary bromide excretion with mortality

	All KTRs (*n* = 693)		Female (*n* = 299)		Male (*n* = 394)	
	HR (95% CI)	*P* value	HR (95% CI)	*P* value	HR (95% CI)	*P* value
Model 1	0.75 (0.57, 0.99)	0.04	0.54 (0.36, 0.80)	0.003	1.01 (0.68, 1.51)	0.9
Model 2	0.71 (0.54, 0.94)	0.02	0.47 (0.31, 0.72)	<0.001	1.03 (0.69, 1.55)	0.9
Model 3	0.69 (0.52, 0.92)	0.01	0.45 (0.30, 0.70)	<0.001	1.00 (0.66, 1.51)	0.9
Model 4	0.67 (0.50, 0.90)	0.008	0.45 (0.29, 0.72)	0.001	0.99 (0.65, 1.51)	0.9

HRs are presented per doubling of 24-h urinary bromide excretion. Total number of events: 150 (female: 61; male: 89).

Model 1: adjusted for age and sex and body surface area; Model 2: additionally adjusted for eGFR (_creatinine + cystatin C_), urinary protein excretion, and time since transplantation; Model 3: additionally adjusted for baseline smoking and alcohol; Model 4: additionally adjusted for hypertension and diabetes.

Abbreviations: CI, confidence interval; eGFR, estimated glomerular filtration rate; HR, hazard ratio; KTR, kidney transplant recipient.

**FIGURE 1:**
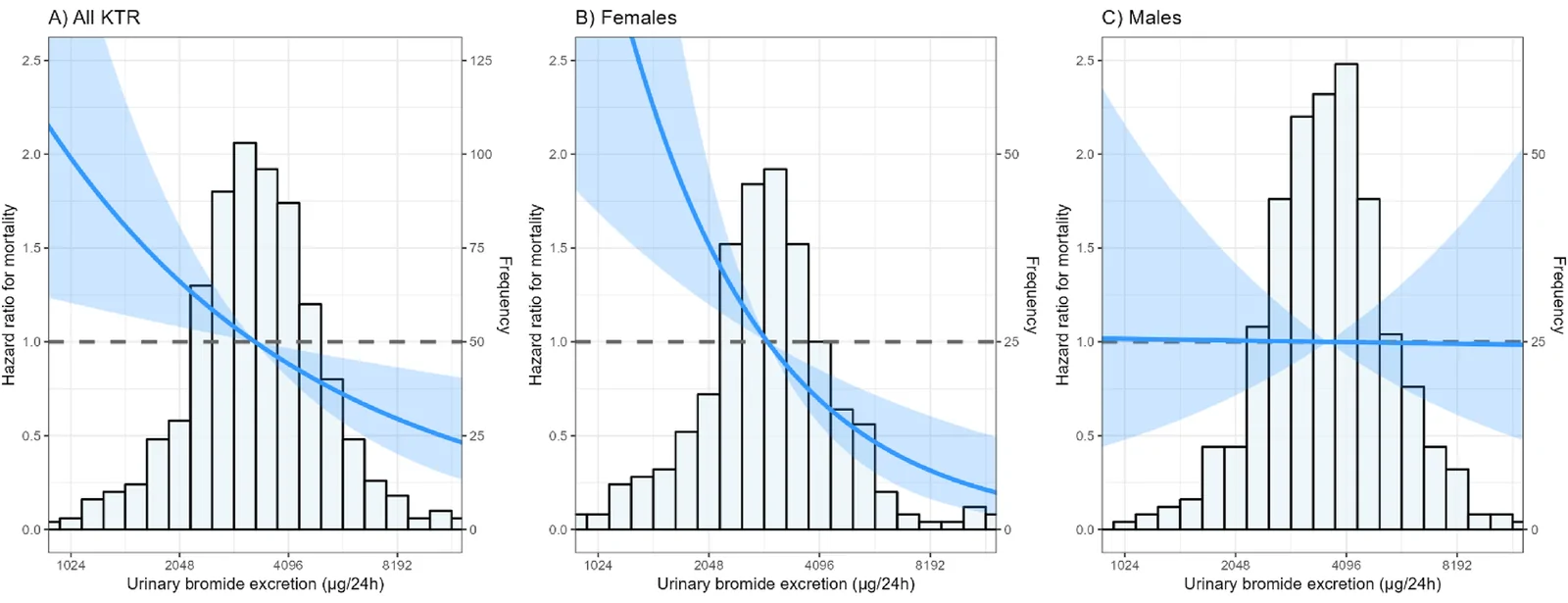
Visual representation of the association of urinary bromide excretion with all-cause mortality according to sex. Analyses adjusted for age, sex, body surface area, eGFR, urinary protein excretion, time since transplantation, smoking status, alcohol intake, hypertension, and diabetes. The median values are used as the reference point. eGFR, estimated glomerular filtration rate; KTR, kidney transplant recipient.

In sensitivity analyses, we further adjusted for total energy intake, outliers by excluding the highest fifth percentile of bromide excretion values, water intake, animal protein intake, and physical activity (Table 4). In the total KTR group, the association between urinary bromide excretion and mortality remained materially unchanged and consistently statistically significant across all sensitivity models (HRs ranging from 0.55 to 0.69). Among female KTRs, none of the sensitivity analyses materially altered the results, as statistical significance was consistently maintained across all models, whereas among male KTRs, none of the sensitivity analyses demonstrated a statistically significant association.

**TABLE 4:** Sensitivity analyses of the Cox regression analyses of 24-h urinary bromide excretion with mortality

	Total (*n* = 693)		Female (*n* = 299)		Male (*n* = 394)	
	HR (95% CI)	*P* value	HR (95% CI)	*P* value	HR (95% CI)	*P* value
Base	0.67 (0.50, 0.90)	0.008	0.45 (0.29, 0.72)	0.001	0.99 (0.65, 1.51)	0.97
Base without outliers^1^	0.55 (0.40, 0.77)	<0.001	0.48 (0.29, 0.79)	0.005	0.66 (0.41, 1.06)	0.08
Base + energy intake	0.68 (0.50, 0.91)	0.01	0.42 (0.28, 0.72)	0.001	1.01 (0.66, 1.54)	0.9
Base + water intake	0.69 (0.51, 0.93)	0.02	0.47 (0.30, 0.75)	0.002	1.02 (0.67, 1.56)	0.9
Base + animal protein intake	0.67 (0.50, 0.90)	0.009	0.47 (0.29, 0.76)	0.002	0.97 (0.64, 1.48)	0.9
Base + FT4 + TSH	0.67 (0.50, 0.91)	0.009	0.45 (0.29, 0.72)	0.001	1.01 (0.66, 1.54)	0.9
Base + physical activity	0.67 (0.50, 0.90)	0.008	0.52 (0.34, 0.80)	0.004	0.99 (0.65, 1.51)	0.9
Base + chloride excretion	0.64 (0.44, 0.95)	0.03	0.41 (0.21, 0.82)	0.012	1.01 (0.61, 1.67)	0.9

Abbreviations: CI, confidence interval; eGFR, estimated glomerular filtration rate; FT4, free thyroxine; HR, hazard ratio; TSH, thyroid-stimulating hormone.

Base model is adjusted for age, sex, body surface area, eGFR (_creatinine + cystatin C_), urinary protein excretion, time since transplantation, smoking status, alcohol intake, hypertension, and diabetes.

1 Given the skewed distribution, outliers are defined as the highest 5th percentile.

In additional sensitivity analyses, further adjustment for RAAS inhibitor use [HR: 0.67 (95% CI: 0.50, 0.89), *P* = 0.007] or protein intake [HR: 0.68 (95% CI: 0.50, 0.92), *P* = 0.01] did not materially alter the association between urinary bromide excretion and mortality in the total population or in sex-stratified analyses (Supplemental Table 4). When creatinine clearance was used instead of eGFR as a measure of kidney function, the association remained significant in females [HR: 0.53 (95% CI: 0.34, 0.83), *P* = 0.007], whereas in the total population the association remained in the same direction but no longer reached statistical significance [HR: 0.75 (95% CI: 0.56, 1.00), *P* = 0.06]. No significant associations were observed in males in any of these additional analyses.

## Discussion

In this study, urinary bromide intake was positively associated with dietary intake, including energy, animal protein, and water intake, with notable differences between males and females. Overall, lower bromide intake was associated with a higher risk of all-cause mortality in KTRs. Notably, this association appeared to be sex-specific. Lower urinary bromide excretion was associated with increased mortality in female KTRs, whereas no significant association was observed in male KTRs. These findings suggest a potential sex-specific relationship between bromide status and long-term survival after kidney transplantation.

In female KTRs, this association appeared approximately linear across the observed range of urinary bromide excretion, with lower excretion associated with higher mortality risk and higher excretion associated with lower risk. This suggests that variation in bromide status within the physiological range may modulate risk, rather than reflecting a simple deficiency or toxicity threshold.

Direct comparison of the observed association between urinary bromide excretion and mortality with previous studies is limited because, to our knowledge, no previous longitudinal population-based studies have investigated this association. Previous human studies have primarily focused on bromide exposure, dietary determinants, pharmacokinetics, and the adverse effects of excessive exposure [3,[10], [11], [12],^20^]. The present findings, therefore, extend the available evidence by suggesting that differences in bromide exposure at levels below those associated with toxicity may also be relevant to long-term health outcomes.

Although bromide has historically been regarded primarily as an ultratrace element and a potential toxicant, emerging evidence indicates that it fulfills specific biochemical functions essential for tissue structure and immune defense [[10], [11], [12]]. Experimental evidence indicates a physiological role in peroxidasin-mediated collagen IV cross-linking and basement membrane integrity [4,5]. However, whether these mechanisms contribute to the association between urinary bromide excretion and mortality observed in the present study remains unknown.

To explore potential mechanisms underlying the observed association between low bromide intake and mortality, we conducted sensitivity analyses adjusting for additional variables that could influence bromide excretion or factors that are part of common pathways of bromide metabolism. To explore whether the association between urinary bromide excretion and mortality was independent of dietary and lifestyle factors that influence bromide levels, we conducted sensitivity analyses adjusting for energy, protein, and water intake, as well as physical activity. Despite additional adjustment for these dietary and lifestyle factors, the associations remained statistically significant, suggesting that they were not explained by differences in overall water intake, energy intake, animal protein intake, or physical activity, but rather reflect an independent relationship between urinary bromide excretion and mortality.

Urinary chlorine excretion was included, given its close physiological relationship with renal bromide handling. Bromide and chloride share common transport pathways in the kidney, including the sodium-chloride cotransporter in the distal convoluted tubule and the chloride/bicarbonate exchanger in the proximal tubule and intercalated cells of the collecting duct [3,35]. In addition, the interplay between chloride and bromide is relevant in the context of the innate immune system, as hypochlorous acid plays a key role in immune defense [36,37]. The association remained significant after this adjustment, suggesting that it was not driven by shared renal handling or immune-related pathways of chloride and bromide. In addition, we adjusted for circulating thyroid hormone levels because bromide and iodide share similar biochemical pathways and compete for uptake via the sodium-iodide symporter in the thyroid gland [38,39]. Despite this adjustment, the association remained statistically significant, suggesting that it was not explained by interference with thyroidal iodide handling or related hormonal pathways.

To date, regulatory guidance has focused primarily on preventing excessive bromide exposure. The EFSA has set a tolerable daily intake of 0.4 mg/kg body weight per day and an acute reference dose of 0.4 mg/kg body weight per day for bromide, based on human and animal toxicological data [3]. This advice is focused on the critical effects of bromide in experimental animal studies on the thyroid and central nervous system. However, several human studies in healthy volunteers suggest that daily doses in the range of 1–9 mg/kg/d are well tolerated and cause no detectable endocrine disruption [40,41]. However, these studies did not examine long-term outcomes. Of note, the maximum bromide intake per kilogram of body weight in the current cohort was 0.29 mg/kg body weight per day, which is below the tolerable daily intake as suggested by the EFSA. This suggests that the associations with mortality are unlikely to be driven by toxic effects of excessive bromide, though the potential consequences of relatively low intake are not defined by current guidelines.

Recently, EFSA performed analyses of 46,965 bromide concentrations from foods collected in 29 European countries between 2013 and 2022, which showed the highest mean concentrations of bromide in the category “coffee, cocoa, tea and infusions,” particularly in dried flowers and herbal infusion materials from leaves and herbs [3]. Historically, average dietary intake in Europe was estimated at 7 to 9 mg/d during the 1970s, with higher levels in populations consuming foods treated with fumigation agents [42]. As many of these sources have declined, current bromide intake may be lower. Population studies suggest that differences in urinary bromide excretion are largely driven by dietary habits [20]. In East Asian populations, higher urinary bromide levels have been associated with greater consumption of marine products, including sea algae, fish, and shellfish, whereas inverse associations have been reported with consumption of cereals and potatoes [20]. Similarly, marked differences in urinary bromide concentrations between Japanese and Chinese populations have been attributed partly to differences in dietary habits [43]. Differences in dietary patterns between these East Asian populations and the Netherlands should be considered when comparing these findings, particularly as consumption of marine products is relatively limited in the Dutch population [44]. This may partly explain why the dietary determinants of urinary bromide excretion identified in our KTR population differ from those reported previously. Although nuts, particularly Brazil nuts, have been reported as dietary sources of bromide [3], nut intake in our population was very low, limiting our ability to meaningfully investigate the association between nut consumption and urinary bromide excretion. Given the potential nutritional benefits of nut enrichment in KTRs [45], future intervention studies increasing nut consumption may provide an opportunity to prospectively investigate its effect on urinary bromide excretion.

In this study, bromide excretion was positively associated with energy, animal protein, and water intake, with notable differences between sexes. Specifically, associations of bromide excretion with energy and animal protein intake were stronger in females. The biological basis of these sex-specific differences remains unclear. Potential explanations may include differences in dietary patterns, body composition, renal handling, or hormonal regulation of bromide metabolism. However, additional analyses adjusting for dietary factors, physical activity, thyroid function, and urinary chloride excretion did not explain the observed sex-specific association. Because bromide, similar to other halide ions, is distributed throughout the extracellular fluid compartment, sex differences in body composition and total body water may contribute to differences in bromide distribution and absolute urinary excretion between females and males [3,46]. Although the underlying mechanisms remain to be elucidated, the observed sex-specific associations highlight the importance of considering sex as a potential modifier of bromide status and health outcomes. Further studies specifically designed to investigate sex differences in bromide metabolism and kinetics are therefore warranted.

This study has several strengths. To our knowledge, this is the first study to examine the association between dietary bromide intake and mortality in KTRs. Other strengths of our study are the long follow-up and the large study population. In addition, extensive data collection of many demographic, dietary, and laboratory parameters enabled adjustment for many potential confounders. Stratification by sex revealed a novel, clinically important sex-specific association between low bromide intake and mortality. However, there are some limitations of this study that should be considered when interpreting these findings. First, the observational nature of the study limits the ability to establish causality. The potential for exposure misclassification was limited by analyzing urinary bromide excretion as a continuous variable rather than categorizing participants according to arbitrary cut-offs for adequate or inadequate intake, for which established reference values are currently unavailable. Furthermore, mortality status was obtained from governmental registration data, minimizing the likelihood of outcome misclassification. Furthermore, this study was carried out in a center with a dominantly White population, necessitating caution when generalizing our findings to individuals from diverse ethnic backgrounds and with different dietary patterns. In addition, the generalizability of our findings beyond this specific KTRs cohort, including to the general population or other patient groups, cannot be determined from the present study. Although participants received detailed instructions and completed FFQs were carefully reviewed, underreporting or overreporting and socially desirable responses cannot be fully excluded. In addition, dietary bromide intake could not be directly estimated from the FFQ because bromide concentrations were not included in the NEVO 2006 food composition table. Consequently, absolute dietary bromide intake and the contribution of individual foods to total bromide intake could not be determined. This limits the interpretation of the analyses of dietary determinants, as observed associations with urinary bromide excretion cannot be translated into the quantitative contribution of specific foods or dietary components to bromide intake. Urinary bromide excretion was assessed from a single 24-h urine collection, which does not account for intraindividual variation over time and may therefore adversely affect its predictive properties. Accounting for intraindividual variability through repeated measurements has been shown to strengthen associations between biomarkers and subsequent outcomes, with greater benefit expected with increasing intraindividual variation [47,48]. The observed association with a single measurement is therefore more likely an underestimation of the association that could be observed if repeated measurements were used than an overestimation. However, the relatively long biological half-life of bromide of ∼12 d may reduce short-term day-to-day variation in urinary bromide excretion [3,19]. Finally, although our analyses partially allowed us to assess certain pathways, we were unable to investigate other mechanisms.

In conclusion, our findings indicate that low bromide intake was associated with an increased risk of all-cause mortality in female KTRs, underscoring a potential sex-specific vulnerability. Future research should focus on a better understanding of the underlying mechanisms and the possible determination of an RDA for bromide intake.

## Author contributions

The authors’ responsibilities were as follows – YvdV, AP, SJLB: designed the research and primarily responsible for the final content; YvdV, AP: conducted the research, analyzed the data or performed statistical analysis, and wrote the manuscript; GR, DJT: provided essential reagents or materials; YvdV, CSED, DK, DJT, EC, GR, CA, CFMF, SJLB: reviewed and edited the manuscript; and all authors: read and approved the final manuscript.

## Data availability

Data described in the manuscript, code book, and analytic code will be made available upon request.

## Declaration of Generative AI and AI-assisted Technologies in the Writing Process

The authors declare that no generative AI or AI-assisted technologies were used in the writing of this manuscript.

## Funding

The establishment of this cohort and its underlying biobank, known as the TransplantLines Food and Nutrition Biobank and Cohort Study (TxL-FN), trial registration number NCT02811835, was funded by the Top Institute Food and Nutrition, grant number A-1003.

## Conflict of interest

The authors declare no conflicts of interest.
