# Supplementary material for: Low Dietary Bromide Intake and Sex-Specific Mortality in Kidney Transplant Recipients
Source: Curr Dev Nutr. 2026 Sep 25;10(10):109543. doi: 10.1016/j.cdnut.2026.109543 (PMC13698046; doi:10.1016/j.cdnut.2026.109543)
Supplement: Supplementary file 1 [file mmc2.pdf]

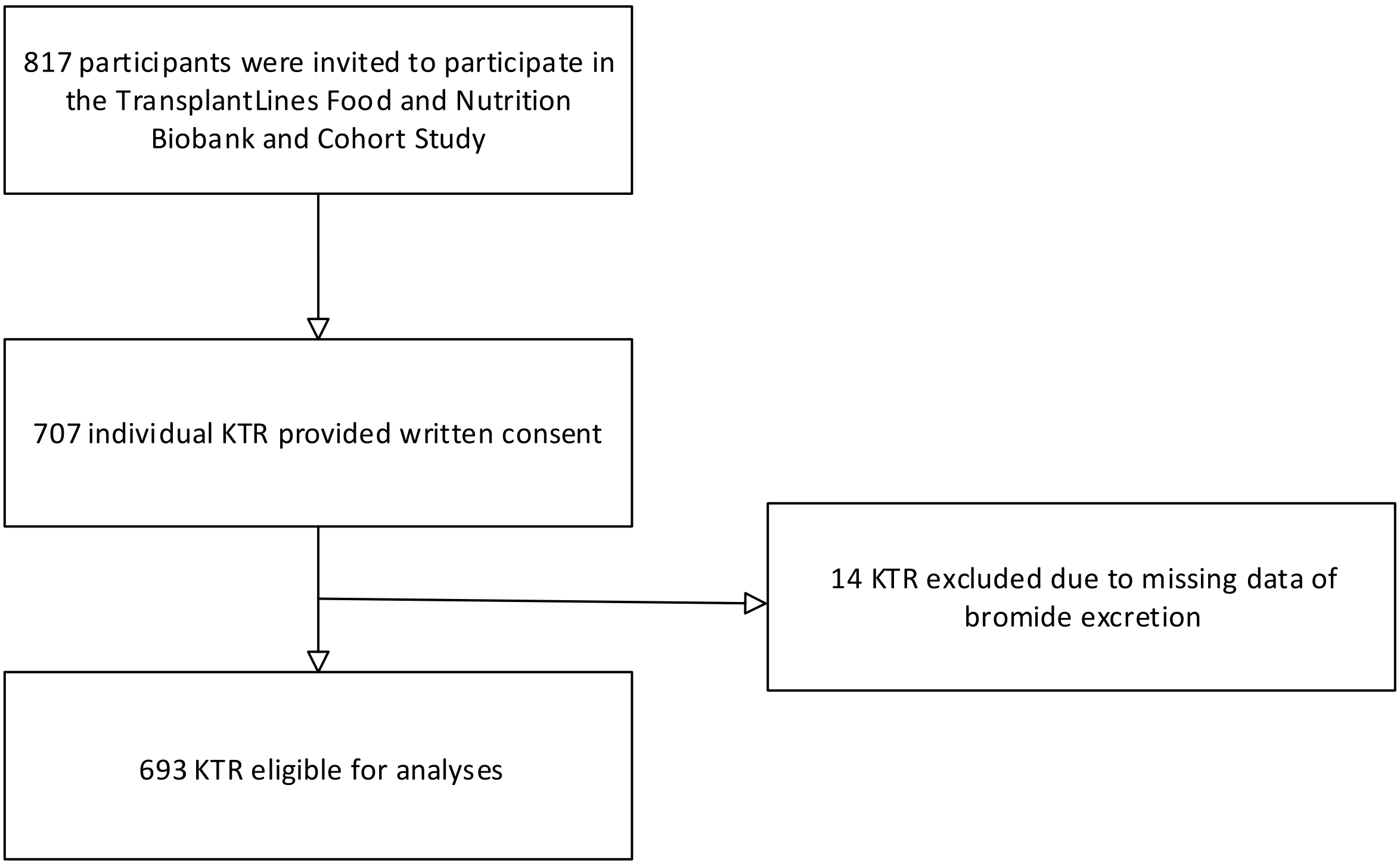
817 participants were invited to participate in the TransplantLines Food and Nutrition Biobank and Cohort Study
707 individual KTR provided written consent
14 KTR excluded due to missing data of bromide excretion
693 KTR eligible for analyses
