# Supplementary material for: Low Dietary Bromide Intake and Sex-Specific Mortality in Kidney Transplant Recipients
Source: Curr Dev Nutr. 2026 Sep 25;10(10):109543. doi: 10.1016/j.cdnut.2026.109543 (PMC13698046; doi:10.1016/j.cdnut.2026.109543)
Supplement: Supplementary file 2 [file mmc1.docx]

**Supplemental materials**

**Title: Low Dietary Bromide Intake and Sex-Specific Mortality in Kidney Transplant Recipients**

**First author:**

Yvonne van der Veen

Department of Internal Medicine, Division of Nephrology

University Medical Center Groningen

9713 GZ Groningen, the Netherlands

Phone Nr: +31625649962

Email: y.van.der.veen@umcg.nl

**Supplemental Figure 1**. Study Flowchart

**Supplemental Table 1**. Strobe statement checklist

| **Section/Topic** | **Item No** | **Recommendation** | **Reported on Page No** |
| --- | --- | --- | --- |
| **Title and abstract** | 1 | (*a*) Indicate the study’s design with a commonly used term in the title or the abstract | 1 |
|  |  | (*b*) Provide in the abstract an informative and balanced summary of what was done and what was found | 3 |
| **Introduction** | | | |
| Background/rationale | 2 | Explain the scientific background and rationale for the investigation being reported | 5 |
| Objectives | 3 | State specific objectives, including any prespecified hypotheses | 5,6 |
| **Methods** | | | |
| Study design | 4 | Present key elements of study design early in the paper | 7,8 |
| Setting | 5 | Describe the setting, locations, and relevant dates, including periods of recruitment, exposure, follow-up, and data collection | 7,8 |
| Participants | 6 | (*a*) *Cohort study*—Give the eligibility criteria, and the sources and methods of selection of participants. Describe methods of follow-up | 7 |
|  |  |  |  |
| Variables | 7 | Clearly define all outcomes, exposures, predictors, potential confounders, and effect modifiers. Give diagnostic criteria, if applicable | n/a |
| Data sources/measurement | 8* | For each variable of interest, give sources of data and details of methods of assessment (measurement). Describe comparability of assessment methods if there is more than one group | 7-10 |
| Bias | 9 | Describe any efforts to address potential sources of bias | 9,11 |
| Study size | 10 | Explain how the study size was arrived at | 10 |
| Quantitative variables | 11 | Explain how quantitative variables were handled in the analyses. If applicable, describe which groupings were chosen and why | 7,10,11 |
| Statistical methods | 12 | (*a*) Describe all statistical methods, including those used to control for confounding | 9-11 |
|  |  | (*b*) Describe any methods used to examine subgroups and interactions | 10,11 |
|  |  | (*c*) Explain how missing data were addressed | 10 |
|  |  | (*d*) *Cohort study*—If applicable, explain how loss to follow-up was addressed | 9,10 |
|  |  | (*e*) Describe any sensitivity analyses | 10,11 |
| **Section/Topic** | **Item No** | Recommendation | n/a |

| **Results** | | | |
| --- | --- | --- | --- |
| Participants | 13* | (a) Report numbers of individuals at each stage of study—eg numbers potentially eligible, examined for eligibility, confirmed eligible, included in the study, completing follow-up, and analysed | 12, supplemental materials |
|  |  | (b) Give reasons for non-participation at each stage | 7, supplemental materials |
|  |  | (c) Consider use of a flow diagram | Supplemental materials |
| Descriptive data | 14* | (a) Give characteristics of study participants (eg demographic, clinical, social) and information on exposures and potential confounders | 12, supplemental materials |
|  |  | (b) Indicate number of participants with missing data for each variable of interest | Supplemental materials |
|  |  | (c) *Cohort study*—Summarise follow-up time (eg, average and total amount) | 12 |
| Outcome data | 15* | *Cohort study*—Report numbers of outcome events or summary measures over time | 12-14 |
|  |  |  |  |
|  |  |  |  |
| Main results | 16 | (*a*) Give unadjusted estimates and, if applicable, confounder-adjusted estimates and their precision (eg, 95% confidence interval). Make clear which confounders were adjusted for and why they were included | 12-14 |
|  |  | (*b*) Report category boundaries when continuous variables were categorized | 12-14 |
|  |  | (*c*) If relevant, consider translating estimates of relative risk into absolute risk for a meaningful time period | n/a |
| Other analyses | 17 | Report other analyses done—eg analyses of subgroups and interactions, and sensitivity analyses | 13,14 |
| **Discussion** | | | |
| Key results | 18 | Summarise key results with reference to study objectives | 15 |
| Limitations | 19 | Discuss limitations of the study, taking into account sources of potential bias or imprecision. Discuss both direction and magnitude of any potential bias | 18,19 |
| Interpretation | 20 | Give a cautious overall interpretation of results considering objectives, limitations, multiplicity of analyses, results from similar studies, and other relevant evidence | 15-19 |
| Generalisability | 21 | Discuss the generalisability (external validity) of the study results | 15-17 |
| **Other Information** | | | |
| Funding | 22 | Give the source of funding and the role of the funders for the present study and, if applicable, for the original study on which the present article is based | 20 |

| **Supplemental Table 2**. Proportion of missing data used for imputations by variable | | | | |
| --- | --- | --- | --- | --- |
| **Variable** | | **N (total)** | **N missing** | **% missing** |
| Urinary bromide excretion | | 693 | 0 | 0 |
| **Clinical characteristics** | |  |  |  |
|  | Age at visit | 693 | 0 | 0 |
|  | Sex | 693 | 0 | 0 |
|  | Body surface area | 693 | 0 | 0 |
|  | Body mass index | 693 | 0 | 0 |
|  | Hypertension | 693 | 0 | 0 |
|  | Diabetes | 693 | 0 | 0 |
|  | Current smoking | 647 | 46 | 6.6 |
|  | Time after transplantation | 693 | 0 | 0 |
| **Laboratory measurements** | |  |  |  |
|  | eGFR | 690 | 3 | 0.43 |
|  | Urinary protein excretion | 693 | 0 | 0 |
|  | Urinary chloride excretion | 693 | 0 | 0 |
| **Dietary intake** | |  |  |  |
|  | Energy intake | 625 | 68 | 9.81 |
|  | Water intake | 632 | 61 | 8.80 |
|  | Dairy | 630 | 63 | 9.09 |
|  | Coffee | 632 | 61 | 8.80 |
|  | Tea | 632 | 61 | 8.80 |
|  | Total plant protein intake | 631 | 62 | 8.94 |
|  | Total animal protein intake | 631 | 62 | 8.94 |
| **Physical activity** | |  |  |  |
|  | SQUASH Score | 693 | 0 | 0 |
| Urinary bromide excretion was complete by definition, as participants with missing values were excluded prior to analysis. Abbreviations: eGFR, estimated glomerular filtration rate; SQUASH, Short Questionnaire to Assess Health-enhancing physical activity | | | | |

| **Supplemental Table 3.** Sex-stratified patient characteristics of KTR according to median urinary bromide excretion | | | | | | | | | | |
| --- | --- | --- | --- | --- | --- | --- | --- | --- | --- | --- |
|  |  | **Total (n= 693)**  **Median: 3308** | | | **Females (n=299)**  **Median: 2958** | | | **Males (n=394)**  **Median: 3640** | | |
|  |  | **Lower half (n=346)** | **Upper half (n=347)** | **P_difference_** | **Lower half (n=149)** | **Upper half (n=150)** | **P_difference_** | **Lower half (n=197)** | **Upper half (n=197)** | **P_difference_** |
|  | Urinary bromide excretion (µg/24h) | 2567 [2517 – 2937] | 4287 [3731 – 5347] | <0.001 | 2343 [1852 – 2639] | 3712 [3286 – 4633] | <0.001 | 2853 [2435 – 3197] | 4283 [4150 – 5606] | <0.001 |
| **Clinical characteristics** | |  |  |  |  |  |  |  |  |  |
|  | Age at visit (years) | 52 ± 14 | 54 ± 12 | 0.087 | 52.7 ± 13.4 | 53.3 ± 12.3 | 0.71 | 52 ± 14 | 54 ± 12 | 0.094 |
|  | Height (cm) | 171.0 ± 9.6 | 176.0 ± 9.2 | <0.001 | 164.8 ± 7.9 | 167.3 ± 7.2 | 0.004 | 178.0 ± 6.9 | 180.4 ± 7.7 | <0.001 |
|  | Weight (kg) | 76.6 ± 15.6 | 84.2 ± 16.4 | <0.001 | 72.4 ± 15.8 | 75.7 ± 15.8 | 0.08 | 81.8 ± 13.8 | 88.5 ± 15.9 | <0.001 |
|  | Body surface area (m^2^) | 1.88 ± 0.21 | 2.00 ± 0.21 | <0.001 | 1.79 ± 0.19 | 1.84 ± 0.18 | 0.014 | 1.99 ± 0.17 | 2.08 ± 0.19 | <0.001 |
|  | Hypertension (n, (%)) | 123 (35.9%) | 160 (46.1%) | 0.008 | 50 (34.0%) | 49 (32.7%) | 0.90 |  |  |  |
|  | Diabetes (*n*, (%)) | 80 (23.1%) | 82 (23.6%) | 0.95 | 41 (27.5%) | 33 (22.0%) | 0.33 | 40 (20.3%) | 48 (24.4%) | 0.40 |
|  | Current smoking (*n*, (%)) | 37 (11.6%) | 44 (13.4%) | 0.56 | 14 (9.9%) | 11 (7.9%) | 0.72 | 28 (15.6%) | 28 (15.1%) | 1.0 |
|  | Time after transplantation (years) | 6 [2 – 12] | 5 [2 – 12] | 0.96 | 6 [2 – 12] | 5 [2 – 12] | 0.79 | 5 [1 – 11] | 6 [3 -12] | 0.11 |
| **Laboratory measurements** | |  |  |  |  |  |  |  |  |  |
|  | Plasma creatinine (µmol/L) | 140 ± 67 | 136 ± 50 | 0.34 | 132 ± 69 | 121 ± 49 | 0.12 | 151 ± 65 | 143 ± 48 | 0.15 |
|  | Plasma cystatin C (mg/L) | 1.9 ± 0.9 | 1.8 ± 0.7 | 0.089 | 1.9 ± 0.9 | 1.8 ± 0.7 | 0.14 | 2.0 ± 0.8 | 1.9 ± 0.7 | 0.21 |
|  | eGFR (mL/min/1.73m^2^) | 44 ± 20 | 46 ± 18 | 0.34 | 43 ± 21 | 45 ± 18 | 0.53 | 45 ± 19 | 46 ± 18 | 0.61 |
|  | Plasma FT3 (pmol/L) | 4.9 ± 0.9 | 4.9 ± 0.8 | 0.66 | 4.8 ± 0.9 | 4.9 ± 0.7 | 0.30 | 4.9 ± 0.9 | 4.8 ± 0.8 | 0.29 |
|  | Plasma FT4 (pmol/L) | 16.2 ± 3.1 | 16.0 ± 2.7 | 0.55 | 16.4 ± 2.6 | 16.3 ± 3.4 | 0.85 | 15.8 ± 2.8 | 16.1 ± 2.8 | 0.29 |
|  | Plasma TSH (mIU/L) | 1.6 [1.0 – 2.4] | 1.7 [1.1 – 2.6] | 0.03 | 1.6 [1.0 – 2.6] | 1.8 [1.2 – 2.6] | 0.22 | 1.6 [1.0 – 2.3] | 1.6 [1.0 – 2.6] | 0.19 |
|  | Urinary volume (ml/24h) | 2236 ± 741 | 2641 ± 804 | <0.001 | 2205 ± 751 | 2682 ± 828 | <0.001 | 2195 ± 694 | 2674 ± 788 | <0.001 |
|  | Urinary protein excretion (g/24u) | 0.18 [0.00 – 0.31] | 0.22 [0.00 – 0.43] | 0.065 | 0.15 [0.00 – 0.27] | 0.15 [0.00 – 0.32] | 0.48 | 0.21 [0.00 – 0.45] | 0.24 [0.00 – 0.51] | 0.77 |
|  | Urinary chloride excretion (mmol/24h) | 114 ± 37 | 180 ± 59 | <0.001 | 99 ± 29 | 153 ± 54 | <0.001 | 129 ± 42 | 196 ± 57 | <0.001 |
|  | Creatinine clearance (mL/min) | 58 [41 – 77] | 69 [52 – 88] | <0.001 | 57 [39 – 73] | 64 [47 – 80] | 0.01 | 60 [43 – 82] | 72 [54 – 92] | <0.001 |
| **Medication** | | | | |  |  |  |  |  |  |
|  | Prednisolone (*n*, (%)) | 343 (99.1%) | 343 (98.8%) | 1.0 | 148 (99.3%) | 147 (98%) | 0.62 | 195 (99.0%) | 196 (99.5%) | 1.0 |
|  | Calcineurin inhibitor (*n*, (%)) | 208 (60.1%) | 189 (54.5%) | 0.15 | 83 (55.7%) | 85 (56.7%) | 0.96 | 119 (60.4%) | 110 (55.8%) | 0.41 |
|  | Proliferation inhibitor (*n*, (%)) | 287 (82.9%) | 286 (82.4%) | 0.93 | 125 (82.3%) | 124 (82.7%) | 1.0 | 166 (84.3%) | 159 (80.7%) | 0.43 |
|  | RAAS blockade (n, (%)) | 160 (46.2%) | 175 (50.4%) | 0.30 | 58 (38.9%) | 62 (41.3%) | 0.76 | 103 (52.3%) | 112 (56.9%) | 0.42 |
|  | Statin (n, (%)) | 184 (53.2%) | 182 (52.4%) | 0.91 | 82 (55.0%) | 78 (52.0%) | 0.68 | 100 (50.8%) | 106 (53.8%) | 0.61 |
| **Dietary intake ^*^** | |  |  |  |  |  |  |  |  |  |
|  | Energy intake (kcal/day) | 2078 ± 646 | 2277 ± 621 | <0.001 | 1882 ± 589 | 1996 ± 495 | 0.09 | 2285 ± 660 | 2436 ± 628 | 0.028 |
|  | Water intake (ml/day) | 1970 ± 543 | 2158 ± 586 | <0.001 | 1919 ± 465 | 2012 ± 569 | 0.14 | 2023 ± 574 | 2254 ± 602 | <0.001 |
|  | Coffee (grams/day) | 375 [250 – 500] | 500 [375 – 625] | <0.001 | 105 [79 – 141] | 375 [250 – 563] | 0.38 | 375 [250 – 625] | 500 [375 – 750] | 0.001 |
|  | Tea (grams/day) | 250 [107 – 500] | 179 [36 – 375] | <0.001 | 250 [125 – 500] | 250 [125 – 500] | 0.96 | 214 [36 – 375] | 125 [11 – 250] | 0.008 |
|  | Bread (grams/day) | 119 [84 – 155] | 138 [103 – 175] | <0.001 | 105 [79 – 141] | 113 [90 – 142] | 0.12 | 139 [102 – 180] | 147 [111 – 184] | 0.11 |
|  | Vegetables (grams/day) | 87 [46 – 113] | 91 [60 – 136] | 0.05 | 91 [60 – 113] | 95 [61 – 124] | 0.17 | 75 [38 – 109] | 92 [61 – 137] | 0.002 |
|  | Fruit (grams/day) | 119 [65 – 232] | 123 [65 – 232] | 0.48 | 118 [66 – 232] | 169 [88 – 249] | 0.007 | 106 [57 – 211] | 118 [50 – 232] | 0.68 |
|  | Potatoes (grams/day) | 118 [71 – 160] | 124 [84 – 177] | 0.030 | 103 [70 – 144] | 100 [60 – 140] | 0.40 | 131 [90 – 199] | 149 [96 – 186] | 0.63 |
|  | Pasta (grams/day) | 19 [8 – 37] | 19 [12 – 32] | 0.88 | 19 [8 – 26] | 16 [8 – 26] | 0.26 | 24 [12 – 39] | 24 [13 – 39] | 0.88 |
|  | Rice (grams/day) | 16 [5 – 30] | 15 [0 – 32] | 0.26 | 16 [6 – 24] | 15 [4 – 32] | 0.47 | 15 [4 – 32] | 15 [0 – 32] | 0.33 |
|  | Legumes (grams/day) | 2 [0 – 11] | 4 [0 – 13] | 0.29 | 4 [0 – 11] | 0 [0 – 11] | 0.21 | 2 [0 – 11] | 4 [0 – 16] | 0.32 |
|  | Nuts (grams/day) | 0 [0 – 3] | 0 [0 – 4] | 0.95 | 0 [0 – 4] | 1 [0 – 5] | 0.37 | 0 [0 – 3] | 0 [0 – 3] | 0.54 |
|  | Beer (grams/day) | 0 [0 – 22] | 0 [0 – 86] | <0.001 | 0 [0 – 0] | 0 [0 – 0] | 0.94 | 21 [0 – 129] | 27 [0 – 171] | 0.24 |
|  | Wine (grams/day) | 0 [0 – 20] | 0 [0 – 29] | 0.36 | 0.0 [0.0 – 20] | 4 [0 – 31] | 0.15 | 0 [0 – 20] | 0 [0 – 29] | 0.36 |
|  | Cheese (grams/day) | 27 [14 – 45] | 31 [18 – 47] | 0.080 | 30 [15 – 44] | 27 [16 – 47] | 0.78 | 25 [14 – 45] | 33 [17 – 46] | 0.16 |
|  | Egg (grams/day) | 7 [4 – 14] | 14 [6 – 16] | <0.001 | 7 [5 – 14] | 9 [7 – 14] | 0.043 | 8 [4 – 14] | 14 [6 – 21] | 0.001 |
|  | Fish (grams/day) | 10 [3 – 18] | 13 [5 – 22] | 0.019 | 12 [4 – 17] | 12 [4 – 22] | 0.41 | 11 [4 – 23] | 12 [4 – 21] | 0.66 |
|  | Soy products (grams/day) | 0 [0 – 0] | 0 [0 – 0] | 0.70 | 0 [0 – 0] | 0 [0 – 0] | 0.095 | 0 [0 – 0] | 0 [0 – 0] | 0.60 |
|  | Meat products (grams/day) | 92 ± 41 | 99 ± 38 | 0.016 | 83 ± 35 | 92 ± 40 | 0.048 | 99 ± 41 | 104 ± 38 | 0.23 |
|  | Dairy (grams/day) | 124 [41 – 145] | 145 [62 – 145] | 0.005 | 124 [41 – 145] | 138 [62 – 145] | 0.11 | 124 [60 – 145] | 124 [62 – 145] | 0.14 |
|  | Protein intake (grams/day) | 78 ± 20 | 87 ± 19 | <0.001 | 73 ± 18 | 82 ± 19 | <0.001 | 82 ± 20 | 90 ± 20 | 0.001 |
|  | Plant protein intake (grams/day) | 29 ± 10 | 32 ± 10 | <0.001 | 27 ± 8 | 29 ± 9 | 0.028 | 32 ± 10 | 34 ± 10 | 0.055 |
|  | Animal protein intake (grams/day) | 48 ± 15 | 55 ± 15 | <0.001 | 47 ± 14 | 53 ± 16 | 0.001 | 50 ± 15 | 56 ± 14 | 0.001 |
| **Physical activity** | |  |  |  |  |  |  |  |  |  |
|  | SQUASH Score | 4920 [1793 – 7178] | 5280 [2475 – 8640] | 0.024 | 4440 [1680 – 6240] | 4560 [2055 – 6878] | 0.53 | 5360 [1800 – 8220] | 6120 [3045 – 9420] | 0.037 |
| Values are presented as mean ± standard deviation (SD), median [interquartile range (IQR)], or number (percentage) as indicated. Abbreviations: KTR, kidney transplant recipients; eGFR, estimated glomerular filtration rate; hs-CRP, high-sensitivity C-reactive protein; FT3, free triiodothyronine, FT4; free thyroxine; TSH, thyroid-stimulating hormone; RAAS, renin–angiotensin–aldosterone system; SQUASH, Short QUestionnaire to ASsess Health-enhancing physical activity.  * Dietary intake was available in a subset of 631 KTR. | | | | | | | | | | |

| **Supplemental Table 4.** Additional sensitivity analyses of the Cox regression analyses of 24-hour urinary bromide excretion with mortality. | | | | | | |
| --- | --- | --- | --- | --- | --- | --- |
|  | **Total (n=693)** | | **Female (n=299)** | | **Male (n=394)** | |
|  | **HR (95% CI)** | **P-value** | **HR (95% CI)** | **P-value** | **HR (95% CI)** | **P-value** |
| Base | 0.67 (0.50;0.90) | 0.008 | 0.45 (0.29;0.72) | 0.001 | 0.99 (0.65;1.51) | 0.97 |
| Base + RAAS | 0.67 (0.50; 0.89) | 0.007 | 0.46 (0.29; 0.73) | 0.001 | 1.00 (0.66; 1.52) | 0.99 |
| Base + creatinine clearance* | 0.75 (0.56; 1.00) | 0.06 | 0.53 (0.34; 0.83) | 0.007 | 1.09 (0.72; 1.67) | 0.67 |
| Base + protein intake | 0.68 (0.50; 0.92) | 0.01 | 0.47 (0.29; 0.75) | 0.002 | 1.00 (0.66; 1.53) | 0.99 |
| ^*^ creatinine clearance was used as substitute for eGFR.  Base model is adjusted for age, sex, body surface area, eGFR (_creatinine + Cystatin C_), urinary protein excretion, time since transplantation, smoking status, alcohol intake, hypertension and diabetes. Abbreviations: HR: hazard ratio; CI: confidence interval; RAAS: | | | | | | |
